# Metabolic Syndrome, Cognitive Impairment and the Role of Diet: A Narrative Review

**DOI:** 10.3390/nu14020333

**Published:** 2022-01-13

**Authors:** Matina Kouvari, Nathan M. D’Cunha, Nikolaj Travica, Domenico Sergi, Manja Zec, Wolfgang Marx, Nenad Naumovski

**Affiliations:** 1 Discipline of Nutrition and Dietetics, Faculty of Health, University of Canberra, Canberra, ACT 2601, Australia; 2 Functional Foods and Nutrition Research (FFNR) Laboratory, University of Canberra, Bruce, Canberra, ACT 2617, Australia; 3 Food and Mood Centre, IMPACT—The Institute for Mental and Physical Health and Clinical Translation, School of Medicine, Barwon Health, Deakin University, Geelong, VIC 3220, Australia; nikolaj.travica@deakin.edu.au (N.T.); wolf.marx@deakin.edu.au (W.M.); 4 Department of Translational Medicine, University of Ferrara, 44121 Ferrara, Italy; domenico.sergi@unife.it; 5 School of Nutritional Sciences and Wellness, University of Arizona, Tucson, AZ 85721, USA; manjazec@arizona.edu

**Keywords:** metabolic syndrome, dementia, cognitive impairment, cognition, nutrition, healthy aging

## Abstract

Background: This narrative review presents the association between metabolic syndrome (MetS), along with its components, and cognition-related disorders, as well as the potential reversal role of diet against cognitive impairment by modulating MetS. Methods: An electronic research in Medline (Pubmed) and Scopus was conducted. Results: MetS and cognitive decline share common cardiometabolic pathways as MetS components can trigger cognitive impairment. On the other side, the risk factors for both MetS and cognitive impairment can be reduced by optimizing the nutritional intake. Clinical manifestations such as dyslipidemia, hypertension, diabetes and increased central body adiposity are nutrition-related risk factors present during the prodromal period before cognitive impairment. The Mediterranean dietary pattern stands among the most discussed predominantly plant-based diets in relation to cardiometabolic disorders that may prevent dementia, Alzheimer’s disease and other cognition-related disorders. In addition, accumulating evidence suggests that the consumption of specific dietary food groups as a part of the overall diet can improve cognitive outcomes, maybe due to their involvement in cardiometabolic paths. Conclusions: Early MetS detection may be helpful to prevent or delay cognitive decline. Moreover, this review highlights the importance of healthy nutritional habits to reverse such conditions and the urgency of early lifestyle interventions.

## 1. Introduction

Metabolic syndrome (MetS) refers to a cluster of metabolic disorders that increase the risk of developing cardiometabolic disorders and mortality [1]. It is estimated that 20–25% of the global adult population have MetS with similar rates between genders (men: 7–34%/women: 5–22%) [2,3,4]. Aging is one of the major contributors to the growing prevalence of the constellation of cardiovascular and metabolic risk factors that constitute MetS. The latest data from the Centers for Disease Control and Prevention (CDC) suggested that the prevalence of prediabetes or MetS is three times higher in US seniors compared with middle-aged adults [5], while the latest atlas generated by the International Diabetes Federation revealed that one in five adults with type 2 diabetes mellitus (T2DM) are over 65 years old [6].

In the last decade, considerable evidence has indicated that cardiovascular and cerebrovascular disease may share similar underlying mechanisms and risk factors [7]. In particular, chronic metabolic insult may lead to the development of atherosclerosis on the cerebral small vessels, consequently resulting in white matter damage and cognitive dysfunction. The concept of “vascular cognitive impairment”, such as the continuum of cognitive deficits and dementia due to cerebrovascular disease, has been widely accepted as an important cause of cognitive impairment [8]. Relatively recent investigations also suggest that vascular risk factors may also contribute to the onset of sporadic Alzheimer’s disease (AD) [8]. Therefore, beyond the direct aggravating role of MetS in the vascular system, increasing the risk of major cardiovascular events and T2DM, there appears to be an underlying pathophysiological association with neurodegenerative disorders.

Lifestyle modifications, especially dietary habits, are identified as the main therapeutic strategy for the treatment and management of MetS [9]. Plant-based diets characterized by frequent consumption of fruits and vegetables, pulses and legumes, whole-grain products, high-quality protein and fat sourced from fish and seafood, as well as limited intake of refined carbohydrates, sodium and saturated fatty acids, are highly encouraged as part of a healthy dietary pattern [9]. The Mediterranean diet stands among the most well-investigated dietary patterns in the prevention and management of MetS [10]. Similarly, other food patterns with similar dietary compounds, such as dietary approaches to stop hypertension (DASH) and traditional Japanese and Nordic diets, have been linked with beneficial effects on diabetes, insulin sensitivity, blood pressure and lipid profile [11,12,13].

The MetS and conditions associated with cognitive decline share common cardiometabolic pathways, as MetS components can trigger the development and progression of cognitive impairment. Importantly, the risk factors for both MetS and cognitive impairment can be reduced by optimizing nutrition [14]. The clinical manifestations, such as hypercholesterolemia, hypertension, T2DM and increased central body adiposity, are nutrition-related risk factors that can be present during the prodromal period before cognitive impairment [15]. Subclinical deficiencies in essential micronutrients, such as vitamin C, vitamin E and β-carotene, as well as vitamin B12, B6 and folate, have been associated with progressive cognitive decline [16]. Hence, dietary factors are considered a first-line strategy to prevent or potentially delay cognitive decline.

**Figure 1:**
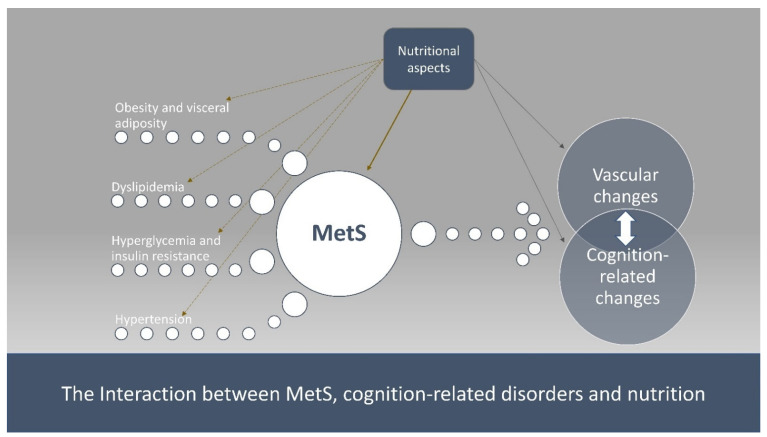
The interaction between MetS, cognition-related disorders and nutrition.

As such, there is a need for data on the interplay between cardiometabolic risk and nutrition in the development of cognitive impairment. Therefore, the aim of the present narrative review was to present (a) the association between MetS, along with its components, and cognition-related disorders, and (b) the potential reversal role of dietary factors or patterns against cognitive impairment by modulating MetS (Figure 1).

## 2. Materials and Methods

An online search in Medline (i.e., Pubmed) and Scopus was conducted and papers that matched with the following keywords were investigated so as to be included in this work: metabolic syndrome, diabetes, dyslipidemia, hypertension, obesity, cognition, cognitive impairment, nutrition, diet, food groups, nutrients, mechanisms and paths. Additionally, the reference lists of the retrieved articles were also considered when these were relevant to the issue examined in the present review but were not found through the basic searching procedure. The search was restricted to studies conducted in humans and published since the year 2000 in the English language so as to include the most recent data from the literature investigating this issue and giving priority to meta-analyses, systematic reviews, clinical trials and well-designed cohorts for more accurate and reliable results.

## 3. Results and Discussion

### 3.1. Cognitive Impairment and Metabolic Syndrome

Dementia is one of the major global health challenges, with almost 50 million people currently living with a diagnosis, and by 2050, this number is expected to increase to 131 million [17]. Dementia is an “umbrella” term that includes several different neurodegenerative conditions, with AD being the most prevalent and contributing up to 70% of all cases, followed by vascular dementia, which accounts for 15–20% of all cases [17]. Although the exact etiology of dementia is still unknown, several metabolic disturbances (i.e., prediabetes, T2DM) have been associated with a modest increased risk of cognitive dysfunction across all cognitive domains [18]. In a meta-analysis of longitudinal studies, a non-significant pooled association between MetS and incident dementia and AD emerged, and MetS was significantly associated with an increased risk of vascular dementia [19]. Although the mechanisms linking MetS with cognitive impairment are not well understood, the current evidence indicates an increased dementia risk in people with T2DM, prediabetes and MetS, with common characteristics between conditions including impaired glycemic control, abnormal lipidemic profile and visceral adiposity [20]. All of these conditions are encompassed under the umbrella of adiposity or obesity-induced cognitive impairment. To date, several meta-analyses of observational studies (Table 1) examined the association between MetS and its components with the onset or progression of cognitive disorders (i.e., mild cognitive impairment, all-cause dementia, vascular dementia, AD), indicating a clear and representative relationship.

#### 3.1.1. Adiposity and Cognitive Impairment

Accumulating evidence exists regarding the detrimental effect of adiposity on the central nervous system, consequently resulting in cognitive impairment, including in the domains of attention, executive function, decision making and verbal fluency [31]. In particular, obesity almost doubles the risk of AD [22], while obesity in midlife predicts a greater risk of all-cause dementia in later life [27]. Furthermore, visceral adiposity is also associated with insulin resistance, which, in turn, reduces capillary reactivity and cerebral blood flow, which is a marker of optimal neuronal activity [32]. In contrast, the extent to which overweight and obesity are risk factors for incident dementia seems to differ between midlife and later life [27], indicating a potential cascade of events related to the chronological onset of obesity [33]. Nevertheless, one of the major problems is that overweight and obese individuals at various life stages often live with other cardiometabolic co-morbidities that are associated with an increased risk of developing dementia [27,34]. The primary genetic risk factor for the development of late-onset AD, namely, apolipoprotein E (ApoE), is also associated with an increased risk of developing MetS [35]. In addition, emerging evidence indicates the importance of obesity-related systemic inflammation [36]. In particular, adipose tissue releases proinflammatory cytokines, such as interleukin-6 (IL-6), and inflammation-related proteins, such as C-reactive protein (CRP), resulting in low-grade systemic inflammation [36]. Moreover, inflammation may alter hypothalamic function, and in turn, cognition and mood through dysregulation of the hypothalamic–pituitary–adrenal (HPA) axis, influencing monoaminergic systems [37,38].

#### 3.1.2. Diabetes, Insulin Resistance and Cognitive Impairment

The developed, and increasingly developing countries, are facing a diabetes epidemic, with 90% of diabetic individuals experiencing T2DM in parallel with obesity [39]. Indeed, obesity is strongly linked with metabolic inflammation and lipotoxicity, which are two key mechanisms that can promote insulin resistance, the hallmark of T2DM [40]. Insulin resistance and pancreatic β-cells failure are the drivers of the chronically elevated circulating glucose levels that characterize T2DM. In particular, chronic hyperglycemia and insulin resistance are at the basis of the complications of T2DM, which were thought to affect only the periphery of the human body by promoting neuropathy, nephropathy and vessel damage [41]. Nonetheless, T2DM also negatively impacts the central nervous system, as indicated by its association with the onset and progression of neurodegenerative diseases [42]. In a relatively recent meta-analysis of prospective studies, moderate-to-high-quality evidence demonstrated that T2DM and prediabetes were associated with an increased risk of dementia and cognitive impairment, supporting the paradigm that hyperglycemia and defective glycemic control are pivotal for brain and cognitive health [43]. In support of this, abnormal fasting or impaired glucose tolerance, HbA1c and abnormal fasting insulin levels are associated with a higher risk of dementia [23]. Furthermore, findings of the National Health and Nutrition Examination Survey (NHANES) revealed that impaired glycemic homeostasis, leading to elevated plasma glucose levels, is one of the components of MetS that is more strongly associated with cognitive decline [20].

From a mechanistic perspective, the detrimental effects exerted by hyperglycemia are strictly dependent on the neurotoxic effects elicited by high glucose levels [43]. The brain makes up 2% of a human’s body weight, but despite this, 20% of the body’s glucose requirement is used by the brain [44]. The high glucose demand of the brain is guaranteed by a glucose uptake system independent of insulin. Indeed, the blood–brain barrier (BBB) and neurons can take up glucose via GLUT-1 and GLUT-3 transporters, respectively, which are both insulin-independent glucose transporters [45]. While glucose uptake via mechanisms independent of insulin represents a clear advantage to fulfill the brain glucose demand, it also makes neurons more susceptible to glucose neurotoxicity. However, the BBB does not seem to prevent hyperglycemia from affecting the brain, as demonstrated by the increase in glucose levels in the brain extracellular fluid of diabetic animals [46]. The increase in interstitial glucose levels, in turn, promotes abnormally high levels of glucose into the neurons, thus triggering gluconeurotoxicity [46]. Glucose is also neurotoxic via different mechanisms, including the polyol pathway, the formation of advanced glycation end products (AGEs), oxidative stress and the activation of mitogen-activated protein kinase (MAP) kinases, which were extensively reviewed elsewhere [47]. However, hyperglycemia is not the only driver of cognitive impairment in the context of T2DM. In this regard, insulin resistance has also been linked with the onset and progression of neurogenerative diseases [48]. It cannot be overlooked that besides its metabolic role in the periphery, insulin also acts in the central nervous system to regulate energy balance, glucose metabolism, neuronal function, plasticity, learning and memory [49]. The importance of the role of insulin in the brain is evidenced by the fact that it can enter the nervous system and regulate its function in light of the broad expression of its cognate receptor throughout the brain, which has been reported in the hypothalamus, as well as areas involved in memory function, such as the hippocampus and the prefrontal cortex [50]. Furthermore, in order to target the central nervous system, intranasal administration of insulin exerted beneficial effects on cognitive function in healthy adults, as well as individuals affected by mild cognitive impairment or AD [51]. Thus, in consideration of the fact that the role of insulin in the central nervous system goes well beyond metabolic regulation, it is plausible that defective insulin signaling and insulin resistance may represent a further mechanism that bridges the gap between MetS and cognitive impairment.

Peripheral insulin resistance was associated with cognitive decline [52]. However, insulin resistance is not limited to peripheral tissues, with impaired insulin signaling being reported to affect the brain, as indicated by a decrease in protein kinase B phosphorylation in a variety of animal and cell models of insulin resistance [53]. Insulin resistance may represent a better predictor of memory impairment than elevated blood glucose, thereby providing further support to the role of insulin resistance as a key mechanism driving cognitive decline [54]. Thus, it appears clear that T2DM and AD share common features regarding insulin resistance and impaired brain glucose metabolism, which has led some investigators to refer to this neurodegeneration as type 3 diabetes [55]. Insulin resistance promotes key pathogenetic features of AD, including increased phosphorylation of tau and accumulation of amyloid β, which further support, also from a mechanistic perspective, the impact of defective brain insulin signaling on cognitive impairment [53]. Further support to the nexus between insulin resistance, hyperglycemia and cognitive impairment is provided by the ApoE gene. Not only do ApoE ε4 carriers not respond to intranasal insulin treatment, but this ApoE isoform also impairs cerebral glucose metabolism, assessed by fluorodeoxyglucose positron emission tomography scan and insulin signaling in mice [56].

Thus, impaired glucose metabolism and homeostasis and insulin resistance are at the forefront in linking MetS with cognitive disorders. In light of this, interventions to improve insulin sensitivity and glucose metabolism are emerging and represent promising strategies to improve cognitive function [57].

#### 3.1.3. Hypertension and Cognitive Impairment

Effective screening and management of hypertension are identified as a Class I recommendation for preventing cognitive decline [22]. Elevated blood pressure, especially in midlife, has been associated with the onset and development of dementia and cognitive impairment later in life [58]. In a meta-analysis of prospective epidemiological studies, moderate-quality evidence indicated that midlife hypertension was related to a 1.19-to-1.55-fold excess risk of cognitive impairment [59]. Additionally, midlife systolic blood pressure over 130 mmHg was associated with an increased risk of cognitive impairment [59]. In another recent meta-analysis of twelve randomized controlled clinical trials, lowering blood pressure with antihypertensive agents was significantly associated with a lower risk of incident dementia or cognitive impairment [60]. Several mechanisms were suggested and potentially grouped into three broad categories: action on the concurrent vascular pathology, action on the vascular component of AD pathophysiology and action on non-vascular targets [61]. Furthermore, these mechanisms can include targeting blood–brain barrier dysfunction, which contributes to amyloid-related cerebral angiopathies and reduced total brain volumes, impaired cerebral blood flow and delivery of nutrients/oxygen into the brain accompanied with significantly poorer cognitive performance [62].

#### 3.1.4. Atherogenic Dyslipidemia and Cognitive Impairment

Low levels of HDL cholesterol (HDL-C) and hypertriglyceridemia are key components of the development of MetS. Dyslipidemia contributes to the development of atherosclerotic lesions, leading to microvascular dysfunction, which has been associated with worse cognitive performance [63]. Many AD susceptibility *loci*, such as the APOE variant carriers identified by genome-wide association studies, are also involved in lipid metabolism [64]. In a network analysis of lipoprotein profile and its association with cognitive impairment, both increased triglycerides (TGs) and low HDL-C levels were associated with poor self-rated cognitive performance [65]. The HDL-C and apolipoprotein A-I (ApoA-I) promote the efflux of excess cholesterol via cholesterol transporters, such as the ATP-binding cassette transporter A1 which is involved in the pathogenesis of AD [66]. Interestingly, increased ApoA-I was shown to be associated with a decreased risk of TD2M in males, but not females, who are more likely to develop AD than males [67]. Thereby, disturbances in the metabolism of HDL-C may influence cognition and neuronal growth and repair, and mounting evidence indicates that HDL-C modulates cognitive function in aging and age-related neurodegenerative disorders [63]. The STOP-Dementia cross-sectional study, involving adults over 65 years diagnosed with AD or mild cognitive impairment, revealed a strong relationship between the levels of small-sized HDL particles and mild cognitive impairment [68]. Furthermore, the role of HDL in the cardiovascular systems has been extensively studied and its cardioprotective roles are well established, where HDL particles can be formed in the systemic circulation and the nervous system. Therefore, HDL particles also play a crucial role in the potential targets for the development of small peptides mimicking the HDL as therapeutics for the treatment of AD [69]. Hypertriglyceridemia is also linked with neurodegeneration, yet limited studies exist with non-significant results [25,70]. However, observational studies suggest increased TG levels in the serum of individuals living with AD [71], in addition to being a shared risk factor between the development of dementia and atherosclerotic CVD [72].

### 3.2. The Role of Diet on Cognitive Impairment

#### 3.2.1. Mediterranean-Type Dietary Pattern

Adherence to the Mediterranean dietary pattern is inversely associated with the risk of developing dementia and cognitive impairment [73]. Findings from large-scale cohort studies and long-term intervention trials support the protective effect of this pattern against several neurodegenerative disorders [74]. Among the largest prospective observational studies are the Singapore Chinese Health Study (16,948 men and women followed for an average of 20 years) [75], the European Prospective Investigation into Cancer and Nutrition (EPIC-Norfolk, 8009 older individuals with an average of 13 years of follow-up) [76], the EPIC-Spain study (16,169 adults followed for approximately 22 years) [77] and the Coronary Artery Risk Development in Young Adults (CARDIA, with follow-up for up to 20 years) [78]. These studies found that higher adherence to a Mediterranean diet was associated with a significantly lower risk of developing all-cause dementia, AD or mild cognitive impairment.

Similar promising outcomes were also revealed by randomized controlled clinical trials, such as the Mediterranean-DASH Diet Intervention for Neurodegenerative Delay (MIND) study with a dietary intervention that highly overlapped with the typical Mediterranean diet yet gave more focus to foods suggested as neuroprotective due to the inclusion of nutrient-dense foods, such as green leafy vegetables and berries [79]. Additionally, a sub-analysis from the Prevención con Dieta Mediterránea (PREDIMED) study (PREDIMED-NAVARRA) showed an improvement in cognition in both Mediterranean diet groups compared with the control, suggesting that even in individuals with established MetS and increased cardiometabolic risk, the protective effect of this dietary pattern still exists [80]. The PREDIMED-NAVARRA study also demonstrated better cognitive performance in non-ApoE-ε4 carriers, but non-ApoE-ε4 carriers, in the clock drawing test following the Mediterranean diet intervention [81]. Comprehensive meta-analyses have summarized the results of observational and interventional studies on the associations between the Mediterranean diet and cognitive impairment and concluded that dietary patterns like the Mediterranean diet have protective effects (Table 2), suggesting that higher adherence may be associated with a lower risk of mild cognitive impairment and AD. Another meta-analysis revealed that the Mediterranean diet has a beneficial effect on global cognition in older adults [82].

Several mechanistic studies suggested that the Mediterranean diet prevents telomere shortening, which is associated with a reduced risk of many age-related diseases, including cognitive impairment [97]. Other studies suggest that increased adherence is inversely associated with various AD biomarkers [98] and positively affects brain morphology and function [99]. In addition, there are several meta-analyses that document the role of the Mediterranean diet in controlling MetS and its components [100,101,102,103], which in turn could have a preventive effect against cognition-related disorders.

#### 3.2.2. Food Groups and Cognition

Although the overall diet can profoundly affect the brain, accumulating evidence suggests that consumption of specific dietary food groups as a part of the overall diet can improve cognitive outcomes. Therefore, several foods, food groups and beverages have been examined regarding their protective effects on cognitive impairment (Table 2).

##### Fruits and Vegetables

In a Greek sub-analysis of the EPIC study, among the components of the Mediterranean diet, only vegetables exhibited a significant inverse association with cognitive decline [104]. It was established that fruits and vegetables are high in various vitamins, such as folate, and there is a strong association between high folate consumption and cognitive performance [105]. Furthermore, fruits, including citrus fruits and berries and green leafy vegetables, are rich in polyphenols, which favorably impact cognitive well-being [106]. Polyphenols, a diverse group of secondary plant metabolites, seem to exert many neurocognitive benefits related to increased cerebral blood flow, reduced oxidative stress and neuroinflammation, improved neurogenesis and neuroplasticity [107,108]. Relatively recent meta-analyses support the neuroprotective properties of fruits and vegetables and also propose several beneficial properties, mainly due to the action of polyphenols [85,89]. A dose–response analysis revealed that an increment of 100 g per day of fruit and vegetable consumption resulted in a significant reduction in cognitive impairment and dementia risk of around 13% [89]. Simultaneously, fruits and vegetables were discussed for their preventive effects against MetS and its components in terms of their protection against cognition-related disorders [109].

##### Grains

Epidemiological studies that examined the separate role of whole grains on cognitive impairment are limited; however, the hypothesis for their neuroprotective effects may be due to their high phytochemical content [106]. This hypothesis was recently supported via a posteriori defined dietary patterns in the context of large-scale cohort studies. A prospective analysis from the China Health and Nutrition Survey revealed that a “multigrain rice” food pattern—consisting of brown rice, millets, black rice and barley—significantly reduced the risk of cognitive impairment in older Asian people compared with a food pattern characterized by white rice and noodles [110]. Similarly, the Whitehall II prospective cohort study found that a dietary pattern with low consumption of whole grains was associated with higher IL-6 levels, which were associated with accelerated cognitive decline at older ages [111].

Furthermore, the importance of high fiber intake in one’s diet, commonly characterized by a high intake of several whole grains, is well established as a key factor in beneficial cardiometabolic and gastrointestinal health. This is predominately due to the action of specific bacteria in the gut on the production of several metabolites, such as short-chain fatty acids [112], that may influence the beneficial health patterns observed in the microbiota-gut-brain axis. Although there is an increased connection between dietary fiber on overall mental health, only relatively few studies have explored the connection between dietary fiber and cognition, with mixed results [113].

##### Animal and Plant-Based Protein Foods

Fish is the primary dietary source of n-3 polyunsaturated fatty acids, which include docosahexaenoic acid (DHA) and eicosapentaenoic acid (EPA). Previous studies have suggested the beneficial effect of fatty fish consumption and its constituent n-3 fatty acids on brain functioning and neurocognitive development may be through its anti-inflammatory effects, blood pressure reduction and endothelial function enhancement [114]. Additionally, the preventive role of fish against MetS may also imply another mechanism through which this food group prevents cognition-related disorders [115].

Limiting the consumption of meat, particularly highly processed meats, remains a common component of healthy dietary patterns. However, meat and meat products are food sources of essential trace elements zinc and iron in the human diet. Dietary zinc [116] and iron intake [117] have beneficial effects on brain function, playing an essential role in learning and memory. A relatively recent study that analyzed data from the UK Biobank revealed that only processed meat might be a risk factor for dementia. Interestingly, a 50 g/day increment in unprocessed red meat intake was associated with 19% and 30% lower risk of all-cause dementia and AD, respectively [118]. Similarly, a recent meta-analysis of observational studies suggested that red meat (processed and unprocessed) consumption was associated with a higher risk of MetS which implied that this may be an internal mechanism through which lower red meat intake may protect against cognition-related disorders [83].

Eggs have a significant amount of vitamins A, B6, B12, riboflavin, folic acid, choline and iron, all of which may benefit brain function [119]. Limited studies examined the role of eggs in the prevention of cognitive impairment. A case–control study in China suggested that increasing egg intake was associated with a lower likelihood of cognitive impairment, yet the observed odds were not clinically relevant [119]. Another cross-sectional analysis with a similar study sample revealed no significant association of eggs with mild cognitive impairment [120]. Similarly, recent results from the Health and Retirement Study and the Health Care and Nutrition study highlighted that although bivariate analyses show that moderate egg consumers displayed a better cognitive performance at baseline assessment, egg consumption was not associated with cognitive performance when adjusting models for covariates known to have a robust association with cognition [121].

Legumes and soy foods are other polyphenol-rich protein foods suggested for their potential neuroprotective properties [106]. A recent analysis from the NHANES study suggested that increased dietary protein intake from legumes was associated with better cognitive function in adults aged 60 years and older [122]. Even if findings regarding soy foods remain inconclusive, it seems that they may have protective effects. Soy isoflavones are phytoestrogens, which are thought to benefit cognition function through their estrogen-like activity [123]. The results of a recent meta-analysis showed that soy isoflavones may improve cognitive function in adults; however, of the 1386 participants included in the analysis, 1252 were women [123]. In contrast, several observational studies, such as the Honolulu–Asia Aging Study [124] and a study from China [125], revealed that tofu (extracted from soybeans) was associated with poorer cognitive performance. However, these studies have many methodological limitations, such as their observational nature.

Finally, nuts have an optimal fatty acid profile, with a high concentration of monounsaturated and polyunsaturated fatty acids, and can be a good source of n-3 fatty acids (i.e., walnuts), antioxidants and anti-inflammatory compounds [126]. Results from a comprehensive systematic review revealed that no consistent evidence exists to support the view that the regular consumption of nuts has a protective effect on cognition in adults of various ages; however, there were some indications that the intake of walnuts specifically may be associated with better cognitive performance in young, middle-aged and older people [126].

##### Milk and Dairy Products

Many dietary guidelines recommend milk and dairy products for meeting the daily calcium, protein and vitamin B12 intake requirements, as these are important for maintaining optimal health in older people. A meta-analysis that examined the potential relationship between milk consumption (with or without other dairy products) and cognitive function showed that the highest level of milk intake, compared with the lowest intake level (as defined by the original studies), was significantly associated with a lower risk of cognitive impairment [92]. However, a more recent meta-analysis showed no significant association between milk intake and cognitive decline in older individuals, underscoring that it is premature to draw a firm conclusion [127,128]. An analysis from the NHANES study revealed that dietary protein from milk and milk-derived products may negatively affect cognitive function in older people consuming in the highest quartile compared to the lowest [122]. Similarly, results from the prospective Atherosclerosis Risk in Communities (ARIC) study [129] and the Supplémentation en Vitamines et Minéraux Antioxidants (SU.VI.MAX) study [130] revealed that greater milk intake may be associated with a greater rate of cognitive decline or lower verbal memory performance over 20 years. Another matter that is highly discussed is the fact that not all dairy products are the same. Regarding this issue, there is the hypothesis that fermented dairy products may have a protective role on cognitive function, probably due to their high content in probiotics and interaction with the gut-brain axis [131].

##### Oils

The latest meta-analysis on dietary fatty acids and the AD risk revealed only a modest preventive potential of n-3 fatty acids against mild cognitive impairment [132]. However, there are positive results regarding the potential neuroprotective role of extra virgin olive oil polyphenols [133,134] and carotenoids in vegetable oils [135].

##### Coffee and Tea

Coffee is the primary source of caffeine in most populations and contains phenolics and other bioactive compounds with potential beneficial or adverse effects on health. Experimental evidence indicates that caffeinated coffee and caffeine, which readily crosses the blood–brain barrier, may influence the processes associated with AD, suppressing brain amyloid-β production, causing microglia activation, reducing hippocampal pro-inflammatory cytokines, protecting against any dysfunction in the blood–brain barrier and preventing cognitive impairment; however, epidemiological data cannot support a cause and effect relationship [87,136]. In a meta-analysis of observational studies, caffeine intake from coffee and tea was also demonstrated to not be associated with cognitive disorders [137]. However, in an analysis of three large prospective cohort studies, both caffeinated and decaffeinated coffee consumption were inversely associated with deaths attributed to CVD and neurological disorders [138]. This finding is supported by the evidence on other phytochemicals found in coffee (excluding the caffeine), such as chlorogenic and caffeic acids, as well as trigonelline, kahweol and cafestol, that might provide neuroprotective effects [139].

Another hypothesis that was tested is that tea consumption may improve mental performance and reduce the progression of cognitive decline. Several studies observed the association between the consumption of tea and lower cognitive impairment rates [140]. These findings were proposed to be due to tea’s antioxidative and anti-inflammatory effects and its components, such as catechins and theanine, that might contribute to the neuroprotection [141,142]. However, the strength of the association between tea consumption and the risk of cognitive impairment remains uncertain and, in some cases, provides little support for the regular consumption of tea (and coffee) in improving cognitive function [143]. This can also be due to the differences in participants, methodological methods used in the current evidence base, quality and type of tea consumed and the extent of tea consumption on modulating the effects on gut microbiota [144].

### 3.3. Nutrients and Phytochemicals on Cognition

Various micronutrients—primarily folate; vitamins B9, B12 and E; n-3 polyunsaturated fatty acids; as well as non-nutrient phytochemicals—were suggested to improve adult neurogenesis, principally due to their antioxidant and anti-inflammatory properties [145]. Folic acid or folate (vitamin B9) is necessary as a regulator for the development of the central nervous system. In vivo studies have shown that vitamin B9, along with vitamins B6 and B12, plays a critical role in DNA methylation, which is an epigenetic phenomenon in the central nervous system that is critical for the maintenance of adult neurogenesis [146]. Furthermore, all three vitamins play a crucial role in the regulation of homocysteine (Hcy), which was also identified as an independent risk factor for the development of CVD. Additionally, elevated Hcy levels have also been associated with a decline in memory, mild cognitive impairment, several different dementias (including the AD) and psychobehavioral and functional complications [147]. Furthermore, supplementation with folic acid was shown to slow cognitive decline in people with mild cognitive impairment. It appears that the type of folic acid provided in supplements plays an important role in the responses against inflammatory markers [146]. Adequate serum levels of vitamin B12 are well established in playing a major role in proper brain development and cognitive function [146]. Dietary B vitamins also appear to play a key role in AD independently of the ApoE ε4 genotype [148]. Many clinical studies indicated that abnormal vitamin B12 status in the serum is strongly correlated with cognitive impairment and brain atrophy [149], and supplementation with vitamin B12 showed the capacity to reduce the rate of brain atrophy in those diagnosed with MCI [150]. Supplementation with B-vitamins was suggested to prevent cognitive decline, principally through reducing the levels of Hcy, which is a neurotoxic chemical and a risk factor for AD [151]; however, the evidence remains contradictory [152].

Preclinical studies showed that vitamin E can regulate adult neurogenesis due to its antioxidant and anti-inflammatory properties [153]. Nevertheless, in a meta-analysis of randomized clinical trials, the alpha-tocopherol form of vitamin E given to people with MCI did not prevent progression to dementia or improve cognitive performance [153]. However, moderate-quality evidence from a single study showed that alpha-tocopherol may slow functional decline in AD [153]. A more recent meta-analysis (total n = 14,262) produced inconclusive results due to high heterogeneity in the measure of progression to AD [154]. The role of n-3 polyunsaturated fatty acids on cognition was extensively studied. Supplementation trials suggested that their effects on cognition may be beneficial only in certain compromised populations (i.e., with habitual diets low in DHA, ApoE genotype) and not in healthy older people [145,155]. However, results from randomized controlled trials do not support supplementation of n-3 fatty acids in the prevention of cognitive decline [156].

Several observational studies also demonstrated that polyphenol-enriched diets are associated with a reduced risk of cognitive decline [157,158]. This activity was associated with anti-inflammatory properties and via mechanisms such as the modulation of lipid metabolism and gut microbiota function. However, long-term studies on humans had mixed results [159]. For instance, a recent meta-analysis showed that resveratrol has no significant effect on memory and cognitive performance in older people [160]. However, another meta-analysis with young and middle-aged participants provided promising findings regarding the usefulness of polyphenol-rich interventions as an inexpensive approach for enhancing circulation of pro-cognitive neurotrophic factors with beneficial effects appearing to depend on the supplementation protocols [107].

These nutrients may influence cognitive function through numerous mechanistic pathways that impact several symptoms associated with MetS. As such, a number of these nutrients, such as vitamin B9 [161], vitamin B12 [162], vitamin E [163], n-3 fatty acids and polyphenols [164], demonstrated the ability to reduce blood pressure, decrease insulin resistance and reduce blood sugar levels, as well as lower circulating TG levels.

### 3.4. The Gut-Brain Axis and the Role of Dietary Interventions

Emerging experimental and clinical evidence has demonstrated the close interconnection between the gastrointestinal tract and the brain, known as the gut-brain axis [165]. The latest research advances described the importance of gut microbiota in influencing these interactions, namely, through bidirectional signaling to the brain through neural, endocrine, immune and humoral links [166,167]. Hence, clinical and experimental evidence suggests that enteric microbiota has an important impact on the central nervous system through neuroendocrine and metabolic pathways, increasing the risk of dementia and Alzheimer’s disease [168].

Probiotics are live microbes with health benefits when received in adequate amounts, plausibly through their anti-inflammatory or anti-oxidative properties. Probiotics may influence the central nervous system and ameliorate age-associated cognitive deficits [169]. A recent meta-analysis of six clinical trials indicated that probiotics improved cognitive performance in individuals living with AD and MCI [170]. Another meta-analysis that investigated the effects of probiotics, prebiotics and fermented food interventions found no significant improvement in cognitive outcomes, suggesting that further studies are required [165]. Hence current evidence is insufficient, and more reliable evidence from large-scale randomized controlled clinical trials is needed.

Gut dysbiosis, characterized as a disruption to the microbiota homeostasis caused by an imbalance in the microflora, has been associated with various metabolic syndrome symptoms, particularly obesity, hyperglycemia and hypertension [171]. Conversely, probiotic microbes, capable of stimulating gut supporting metabolites, namely, short-chain fatty acids, have been linked with reduced over-eating and obesity, reduced risk of diabetes, hypertension and elevated cholesterol [172]. Based on these findings, interventions targeting the gut may also produce promising results in alleviating the symptoms associated with MetS and, therefore, improve cognition.

## 4. Conclusions

The present narrative review summarized the recent literature on the association between MetS and its components on cognition and neurodegenerative disorders. This review highlights the importance of healthy nutritional habits to reverse such conditions and the urgency of early lifestyle interventions. Recognizing and reducing MetS components may be helpful to prevent or delay cognitive decline.

Further studies are needed to determine whether the early detection of and interventions for MetS can prevent or delay cognitive decline. Future research should focus on populations with increased risk of cognitive decline (e.g., people with MCI or ApoE ε4 genotype) and modifiable risk factors, such as their dietary habits. Focus should also be oriented toward populations of lower socioeconomic status. In addition, the current and future effects of the COVID-19 pandemic and its impact on overall and psychosocial health will require investigation. Up to 50% of people who have died from COVID-19 have had metabolic and vascular co-morbidities [173]. Further, people who have been hospitalized for COVID-19 are more likely to experience cognitive impairments, which is currently considered to be a “long COVID” symptom [174]. Therefore, consideration of COVID-19 infection should be considered from now on to evaluate these interactions and suggest tailor-made prevention and treatment strategies to preserve cognitive function and treat MetS.

## Figures and Tables

**Table 1:** Meta-analyses of epidemiological studies evaluating the association of metabolic syndrome and its components with cognitive impairment.

First Author, Year	Exposure	Outcome	Studies, N	RR (95%CI)
Zuin, M., 2021 [20]	Metabolic syndrome	Alzheimer’s disease	6	1.10 (1.05, 1.15)
Zuin, M., 2021 [21]	Hypertension	Alzheimer’s disease	6	1.05 (1.04, 10.6)
Zuin, M., 2021 [21]	Low HDL-C	Alzheimer’s disease	6	1.07 (1.06, 1.07)
Zuin, M., 2021 [21]	Hypertriglyceridemia	Alzheimer’s disease	6	1.06 (1.05, 1.06)
Zuin, M., 2021 [21]	Obesity in late life	Alzheimer’s disease	6	0.84 (0.74, 0.95)
Yu, J. T., 2020 [22]	Type II diabetes	Alzheimer’s disease	na	1.69 (1.51, 1.89)
Yu, J. T., 2020 [22]	Hypertension	Alzheimer’s disease	na	1.38 (1.29, 1.47)
Xue, M., 2019 [23]	Type II diabetes	Global cognitive decline	20	1.25 (1.12, 1.39)
Xue, M., 2019 [23]	Type II diabetes	Executive function decline	10	1.44 (1.23, 1.69)
Xue, M., 2019 [23]	Type II diabetes	Memory function impairment	10	1.27 (1.16, 1.39)
Xue, M., 2019 [23]	Type II diabetes	Mild cognitive impairment	9	1.49 (1.26, 1.77)
Xue, M., 2019 [23]	Type II diabetes	Dementia	31	1.43 (1.33, 1.53)
Xue, M., 2019 [23]	Type II diabetes	Alzheimer’s disease	24	1.43 (1.25, 1.62)
Xue, M., 2019 [23]	Type II diabetes	Vascular dementia	17	1.91 (1.61, 2.25)
Atti, A. R., 2019 [19]	Metabolic syndrome	Dementia	9	
Atti, A. R., 2019 [19]	Type II diabetes	Dementia	19	
Pal, K., 2018 [24]	Metabolic syndrome	Transition from mild cognitive impairment to dementia	12	2.95 (1.23, 7.05)
Anstey, K. J., 2017 [25]	Low high-density lipoprotein cholesterol	Mild cognitive impairment	2	0.97 (0.75, 1.27)
Anstey, K. J., 2017 [25]	Low high-density lipoprotein cholesterol	Alzheimer’s disease	3	0.78 (0.54, 1.13)
Anstey, K. J., 2017 [25]	Low high-density lipoprotein cholesterol	Vascular dementia	2	1.13 (0.60, 2.14)
Anstey, K. J., 2017 [25]	Low high-density lipoprotein cholesterol	Dementia	2	1.06 (0.71, 1.56)
Anstey, K. J., 2017 [25]	Hypertriglyceridemia	Vascular dementia	2	1.66 (0.68, 4.04)
Pal, K., 2018 [24]	Type II diabetes	Transition from mild cognitive impairment to dementia	12	1.53 (1.20, 1.97)
Li, J. Q., 2016 [26]	Hypertension	Transition from mild cognitive impairment to dementia	7	1.18 (1.10, 1.27)
Li, J. Q., 2016 [26]	Type II diabetes	Transition from mild cognitive impairment to dementia	7	1.52 (1.20, 1.91)
Li, J. Q., 2016 [26]	Hypercholesterolaemia	Transition from mild cognitive impairment to dementia	4	0.48 (0.13, 1.82)
Li, J. Q., 2016 [26]	High body mass index in late life	Transition from mild cognitive impairment to dementia	4	0.85 (0.76, 0.96)
Pedditzi, E., 2016 [27]	Obesity in midlife	Dementia	7	1.41 (1.20, 1.65)
Pedditzi, E., 2016 [27]	Obesity in late life	Dementia	16	0.83 (0.74, 0.94)
Cooper, C., 2015 [28]	Type II diabetes	Transition from mild cognitive impairment to dementia	7	1.65 (1.12, 2.43)
Cooper, C., 2015 [28]	Hypertension	Transition from mild cognitive impairment to dementia	7	1.19 (0.81, 1.73)
Cheng, G., 2012 [29]	Type II diabetes	Alzheimer’s disease	16	1.46 (1.20, 1.77)
Cheng, G., 2012 [29]	Type II diabetes	Vascular dementia	10	2.49 (2.09, 2.97)
Cheng, G., 2012 [29]	Type II diabetes	Dementia	11	1.51 (1.31, 1.74)
Cheng, G., 2012 [29]	Type II diabetes	Mild cognitive impairment	2	1.12 (1.00, 1.45)
Profenno, L. A., 2010 [30]	Obesity in midlife	Alzheimer’s disease	6	1.59 (1.02, 2.48)
Profenno, L. A., 2010 [30]	Type II diabetes	Alzheimer’s disease	8	1.54 (1.33, 1.79)

Abbreviations: na, not available; RR, relative risk; 95%CI, 95% confidence interval.

**Table 2:** Meta-analyses of epidemiological and/or intervention studies evaluating the role of the Mediterranean diet and specific food groups on cognitive impairment.

First Author, Year	Exposure	Outcome	Studies, N	Participants, N	Cases, N	Comparison	RR (95%CI)
Zhang, H., 2020 [83]	Total meat	Cognitive disorders	5	na	na	At least weekly intake vs. other	0.73 (0.57, 0.88)
Bakre, A. T., 2018 [84]	Fish	Dementia	9	40,668	3139	High vs. low	0.80 (0.74, 0.87)
Mottaghi, T., 2018 [85]	Fruits and vegetables	Cognitive impairment	6	17,537	na	High vs. low	0.79 (0.67, 0.93)
Lee, J., 2018 [86]	Milk and dairy products	Cognitive impairment/decline	3	5460	701	High vs. low	1.21 (0.81, 1.82)
Larsson, S. C., 2018 [87]	Coffee	Dementia	4	16,473	2173	Per 1 cup/day	1.01 (0.96, 1.05)
Larsson, S. C., 2018 [87]	Coffee	Alzheimer’s disease	4	308,441	5370	Per 1 cup/day	1.02 (0.96, 1.08)
Liu, X., 2017 [88]	Tea	Alzheimer’s disease	3	5677	249	High vs. low	1.18 (0.84, 1.66)
Liu, X., 2017 [88]	Tea	Cognitive decline	3	7842	1932	High vs. low	0.70 (0.57, 0.88)
Jiang, X., 2017 [89]	Fruits and vegetables	Cognitive impairment and dementia	9	31,104	4583	High vs. low	0.80 (0.71, 0.89)
Wu, L., 2017 [90]	Mediterranean diet score	Mild cognitive impairment	5	24,274	2351	High vs. low	0.83 (0.74, 0.93)
Wu, L., 2017 [90]	Mediterranean diet score	Mild cognitive impairment	5	11,101	1113	Per 1 point increase	0.94 (0.91, 0.98)
Wu, L., 2017 [90]	Mediterranean diet score	Alzheimer’s disease	4	4845	498	High vs. low	0.63 (0.48, 0.82)
Wu, L., 2017 [90]	Mediterranean diet score	Alzheimer’s disease	4	4845	498	Per 1 point increase	0.93 (0.88, 0.97)
Zhang, H., 2016 [91]	Fish	Dementia	3	15,713	1124	Per 1 serving/week	0.95 (0.90, 1.00)
Zhang, H., 2016 [91]	Fish	Alzheimer’s disease	3	16,528	969	Per 1 serving/week	0.88 (0.80, 0.97)
Wu, L., 2016 [92]	Milk	Cognitive disorders	7	10,941	na	High vs. low	0.72 (0.56, 0.93)
Wu, L., 2016 [92]	Milk	Cognitive impairment	5	10,941	na	High vs. low	0.76 (0.50, 1.17)
Wu, L., 2016 [92]	Milk	Dementia	3	10,941	na	High vs. low	0.70 (0.48, 1.02)
Wu, L., 2016 [92]	Milk	Alzheimer’s disease	2	10,941	na	High vs. low	0.63 (0.44, 0.90)
Cao, L., 2016 [93]	Mediterranean diet score	Dementia	3	10,941	na	High vs. low	0.69 (0.57, 0.84)
Psaltopoulou, T., 2013 [94]	Mediterranean diet score	Cognitive impairment	7	8291	1278	High vs. low	0.60 (0.43–0.83)
Sofi, F., 2010 [95]	Mediterranean diet score	Neurodegenerative disorders	4	133,626	na	Per 2 point increase	0.87 (0.81, 0.94)
Sofi, F., 2008 [96]	Mediterranean diet score	Alzheimer’s and Parkinson’s disease	2	133,626	783	Per 2 point increase	0.87 (0.80, 0.96)

Abbreviations: na, not available; RR, relative risk; 95%CI, 95% confidence interval.
